# Family sports environment and emotional regulation in Chinese adolescents: the mediating roles of sports lifestyle and parent–child interaction

**DOI:** 10.3389/fpsyg.2026.1812203

**Published:** 2026-06-04

**Authors:** Zhixing Zhou, Weijian Liu, Jun Xiang

**Affiliations:** School of Physical Education and Health, Zhaoqing University, Zhaoqing, China

**Keywords:** adolescents, emotional regulation, family sports environment, mediating effect, mental health, parent–child relationship, psychological resilience, sports lifestyle

## Abstract

Against the backdrop of increasingly prominent mental health issues among adolescents, insufficient development of the family sports environment, a key external factor associated with emotional regulation, has emerged as a critical but often overlooked determinant of psychological stability and resilience among Chinese youth. In particular, the absence of an active sports lifestyle and structured parent–child interaction patterns may be associated with impaired emotional regulation processes, highlighting the need to examine the associational pathways.

**Objective:**

This study aimed to investigate the association between the family sports environment and emotional regulation abilities among Chinese adolescents and to explore the serial indirect associations of sports` lifestyle and parent–child relationship within a theoretically informed mediation model.

**Methods:**

A cluster sampling survey was conducted among 750 Chinese adolescents. Data were collected using the Family Sports Environment Scale, the Adolescent Emotional Regulation Scale, the Sports Lifestyle Scale, and the Parent–Child Relationship Scale. All variables were measured at a single time point using self-report questionnaires.

**Results:**

(1) No statistically significant differences were observed across gender or age in family sports environment, emotional regulation, sports lifestyle, or parent–child relationship (*p* > 0.05); (2) The family sports environment was significantly and positively associated with emotional regulation (*r* = 0.698, *p* < 0.001). Regression analyses indicated that the total effect of family sports environment on emotional regulation was significant (unstandardized coefficient, *B* = 0.292; *t* = 26.811, *p* < 0.001). while the direct effect also remained significant (standardized coefficient,*β* = 0.207; *t* = 3.829, *p* < 0.001). In addition, the family sports environment was significantly associated with variations in sports lifestyle (*B* = 1.957, *t* = 37.969, *p* < 0.001) and parent–child relationship (*B* = 0.292, *t* = 8.445, *p* < 0.001). Sports lifestyle was significantly associated with variations in parent–child relationship (*β* = 0.226, t = 15.931, *p* < 0.001) and emotional regulation (*β* = 0.203, *t* = 8.400, *p* < 0.001). Moreover, the parent–child relationship was significantly associated with variations in emotional regulation (*β* = 0.524, *t* = 9.175, *p* < 0.001); (3) Sports lifestyle and parent–child relationship both demonstrated significant mediating associations in the relationship between family sports environment and adolescent emotional regulation, consistent with a serial mediation model of associations.

**Conclusion:**

The family sports environment was significantly associated with healthier sports lifestyles among Chinese adolescents and more positive parent–child relationships, and was also indirectly associated with emotional regulation abilities through the sequential mediating effects of these two factors. Sports lifestyles and parent–child relationships function as key intermediary pathways linking the family sports environment to adolescents’ emotional regulation, thereby providing a theoretically informed explanation of the observed associative pathways. From a practical perspective, these findings suggest that optimizing the family sports environment, encouraging parent–child engagement in physical activities, and fostering regular physical activity habits among adolescents may be supportive contextual factors for improving emotional regulation and supporting mental health development. Such strategies may also provide valuable implications for family-based sports guidance and adolescent mental health support practices. However, this study is subject to limitations related to sample representativeness and cross-sectional research design. Future research is encouraged to employ longitudinal designs and incorporate more comprehensive family-context variables to further examine and validate these associations.

## 1 Introduction

In recent years, the relationship between the family sports environment and adolescents’ emotional regulation has received increasing scholarly attention at the intersection of adolescent mental health and physical education research. In 2023, the “Special Action Plan for Comprehensively Strengthening and Improving Student Mental Health Work in the New Era 2023–2025,” jointly issued by the Chinese Ministry of Education and 16 other departments, explicitly emphasized supporting student mental health through the principle of “strengthening the mind through physical activity,” thereby highlighting the potential relevance of physical activity for emotional regulation and stress relief (Chen and Qian, 2025). This framework conceptually links physical activity to adolescent mental health development at the national governance level and provides both institutional support and practical direction for investigating emotional regulation from the perspective of the family sports environment (Yuan et al., 2024). In this context, it becomes particularly important to explore the role of the family sports environment. Specifically, understanding its association with adolescents’ emotional regulation holds considerable practical relevance.

Emotional regulation in adolescents refers to the psychological processes through which individuals perceive, evaluate, and modulate their emotional states (Doron and Anaki, 2024). Its developmental level is closely associated with mental health outcomes and social adaptation (Yılmazer, 2024). Existing research suggests that factors such as parenting styles, peer relationships, and academic stress are significantly related to adolescents’ emotional regulation abilities (Sahi et al., 2023). However, compared with these commonly examined psychosocial determinants, family environmental contexts related to physical activity have received relatively limited scholarly attention. The family sports environment generally refers to a multidimensional construct. It encompasses the material conditions available within the home that support or facilitate physical activity. It also includes the overall exercise-related atmosphere created within the family context. In addition, it reflects the attitudes and behaviors of family members toward physical activity (Lou et al., 2025). Empirical studies have shown that a supportive family sports environment is associated with higher levels of physical activity participation among adolescents, as well as more favorable indicators of mental health outcomes (Li S. et al., 2025; Li Z. et al., 2025; Morris et al., 2007). In a study of Chinese adolescents, Ren and Li (2020) found that social support was significantly associated with physical activity levels and indirectly related to behavioral outcomes through exercise self-efficacy. This suggests that supportive physical activity contexts may also be associated with emotional outcomes through related psychological processes.

From a behavioral health perspective, studies by Cao et al. (2022) and Lu et al. (2020) further indicate that physical activity levels, sedentary behavior, and sleep quality are closely associated with adolescents’ negative emotions and overall mental health indicators. Similarly, Chi and Wang (2022) reported that Chinese adolescents with higher levels of physical activity participation tend to exhibit lower levels of depression and anxiety. Collectively, these findings suggest consistent associations between exercise-related behaviors and adolescents’ emotional health, although most existing research has primarily focused on broader behavioral patterns or general forms of social support rather than specific family-based environmental factors. It is also important to emphasize that, within the Chinese cultural context, the family occupies a central position in adolescents’ daily life organization and behavioral socialization (Zhu et al., 2023; Zhang and Ng, 2022). Parents play a central role in shaping how adolescents allocate their time daily. In addition, parents affect adolescents’ engagement in physical activity through their attitudes and behavioral support. Taken together, these parental influences suggest that the family sports environment constitutes a key contextual factor. This context may be closely linked to the emotional development of adolescents.

Building on this perspective, sports lifestyle and parent–child relationship are examined as potential psychosocial pathways associated with the family sports environment and adolescents’ emotional regulation (rather than implying causal linking). It should be noted that sports lifestyle reflects relatively stable patterns in adolescents’ engagement in, and attitudes toward, physical activity, whereas parent–child relationship refers to the emotional bond and quality of interaction between parents and children (Luo et al., 2025). Existing studies suggest that supportive family physical activity environments are associated with adolescents’ adoption of more active lifestyles and with more positive parent–child interaction patterns (Lisinskiene and Lochbaum, 2019). These factors may, in turn, be associated with adolescents’ emotional regulation abilities. However, there is still a notable lack of systematic empirical research that investigates the potential sequential mediation-based associations among these variables within Chinese adolescent populations. Against the backdrop of increasing academic pressure and the widespread prevalence of sedentary behavior among Chinese adolescents (Shen et al., 2020), further investigation into how the family sports environment is related to adolescents’ emotional regulation through both behavioral and relational pathways may provide important theoretical and practical insights.

Therefore, this study focuses on the family sports environment and examines the associations between sports lifestyle and parent–child relationships as potential mediating variables, including a sequential (chain-mediation) analytical model, in the association between the family sports environment and emotional regulation among Chinese adolescents. In doing so, it aims to advance current understanding by exploring the psychosocial pathways that may be associated with family-based physical activity contexts and adolescents’ emotional regulation, rather than establishing underlying causal mechanisms, building upon and extending prior research.

## 2 Literature review and research hypotheses

### 2.1 Family sports environment and adolescent emotional regulation

The family sports environment is generally defined as the combination of multiple family-related factors, including material conditions that enable adolescents’ participation in physical activity, the overall family atmosphere surrounding physical activity, and family members’ attitudes toward and behaviors related to sports participation (Zheng et al., 2025; Gustafson and Rhodes, 2006). Existing research indicates that a supportive family sports environment is associated with adolescents’ stronger willingness to engage in physical activity and with a greater sense of psychological belonging that is described as being supported through family interaction and supportive behaviors (Zheng et al., 2025). From the perspective of adolescents’ physical and mental development, the family sports environment is reflected not only in the organization of shared family-based physical activities and the creation of an encouraging activity atmosphere, but also in parents’ respect for adolescents’ willingness to participate in sports, their understanding of adolescents’ activity needs, and their sustained support and guidance. Together, these elements constitute an important social context that is associated with adolescents’ engagement in physical activity and may be related to their psychological and emotional development.

Previous research indicates that a supportive family sports environment is associated with higher levels of adolescents’ motivation to engage in physical activity, as well as more favorable emotional regulation outcomes (Zhang et al., 2024). Moreover, empirical findings suggest that higher-quality family sports environments are consistently associated with more favorable emotional regulation outcomes in adolescents (Xie, 2023). For instance, Li et al. (2022) found that a positive home sports atmosphere and a greater diversity of parent–child sport activities were significantly associated with adolescents’ emotional regulation capabilities. In parallel, participation in sports has been linked to enhanced self-confidence and psychological security, which may be relevant to the development of more adaptive emotional regulation strategies (Eime et al., 2013). From a theoretical perspective, Self-Determination Theory posits that supportive social environments contribute to the satisfaction of individuals’ basic psychological needs for autonomy, competence, and relatedness, which are fundamental to positive psychological development (Felber Charbonneau and Camiré, 2020). Within this theoretical framework, the family sports environment can be conceptualized as a positive contextual resource that is theoretically associated with adolescents’ emotional support experiences and may provide behavioral guidance (Zheng et al., 2023).

Collectively, these findings indicate that the family sports environment is not only directly associated with adolescents’ emotional regulation but may also be related to emotional regulation through various interrelated psychological and behavioral pathways. Accordingly, Hypothesis 1 (H1) is formulated as follows: *the family sports environment is significantly associated with emotional regulation among Chinese adolescents*.

### 2.2 Mediating role of sports lifestyle

A sports lifestyle generally refers to relatively stable behavioral patterns and habits shaped by individuals’ cognition of physical activity, including participation frequency and activity preferences (Wintle, 2022). Existing research indicates that the family sports environment is closely associated with the development of adolescents’ sports lifestyles. A supportive family sports climate, parents’ active role modeling in physical activity, and shared parent–child participation in sports are all commonly associated with adolescents’ greater likelihood of engaging in regular exercise and cultivating an active sports-oriented lifestyle (Zheng et al., 2025). For instance, empirical evidence has shown a significant positive correlation between the family sports environment and adolescents’ sports lifestyles. When parents actively engage in sports and encourage their children’s participation, adolescents are more likely to report healthier and more sustained sports lifestyle patterns (Nicholls et al., 2014).

In addition, existing research indicates that sports lifestyles are not only associated with the family sports environment but are also linked to adolescents’ emotional regulation abilities. Adolescents who engage in more active, structured, and consistent sports lifestyles generally tend to demonstrate higher levels of emotional regulation. It is worth noting that regular participation in physical activity has been associated with stress reduction, improved emotional adjustment and emotional adaptability, and is therefore widely discussed as a behavioral factor that may be related to adolescents’ emotional regulation processes (Liu et al., 2024). Overall, these findings suggest that sports lifestyles may serve as an important behavioral context through which adolescents’ emotional regulation is understood to be associated and shaped in correlational terms.

From a theoretical perspective, Self-Determination Theory proposes that environments supportive of physical activity facilitate the satisfaction of adolescents’ basic psychological needs for autonomy, competence, and relatedness in sports participation. These satisfied needs, in turn, are associated with higher levels of intrinsic motivation for physical activity (Morbée et al., 2023). Within this framework, the family sports environment may play a key role in shaping adolescents’ participation in physical activity and the development of their sports-related lifestyle patterns. These lifestyle patterns may subsequently be related to adolescents’ emotional regulation abilities. Collectively, these considerations suggest a potential associative mediating pathway in which the family sports environment is associated with adolescents’ sports lifestyles, which in turn are related to their emotional regulation. Accordingly, Hypothesis 2 (H2) is proposed: *the sports lifestyle mediates the relationship between the family sports environment and emotional regulation among Chinese adolescents*.

### 2.3 Mediating role of parent–child relationship

In this study, parent–child relationship primarily refers to adolescents’ perceptions of emotional closeness and interaction quality with their parents. This construct is reflected in emotional support, communication quality, and perceived relational closeness (Laursen and Collins, 2009; Boele et al., 2019). Previous research has demonstrated a significant positive association between parent–child relationships and emotional regulation among Chinese adolescents. Adolescents who experience more harmonious parent–child relationships are more likely to report stronger emotional support during affective experiences and to develop more adaptive coping tendencies, both of which are associated with enhanced emotional regulation (Aneesh et al., 2024). For example, Brumariu (2015) found that positive parent–child relationships are associated not only with stronger emotional security but also with greater self-awareness and satisfaction in emotional regulation, partly through more effective emotional communication between parents and children. Overall, these findings suggest that the parent–child relationship is an important psychosocial factor associated with emotional regulation among Chinese adolescents.

In addition, scholars have emphasized that the family sports environment, as a context for positive parent–child interaction and the development of health-related behaviors, is closely linked to both adolescents’ sports lifestyles and the quality of parent–child relationships. Joint participation in family sports activities, along with a supportive sporting atmosphere within the household, is frequently linked to adolescents’ more consistent engagement in physical activity and the strengthening of emotional bonds through shared experiences and interaction (Song, 2024). Empirical evidence further indicates significant positive correlations between the family sports environment, adolescents’ sports lifestyles, and parent–child relationships. Through joint exercise participation and behavioral modeling, the family sports environment may be associated with adolescents’ awareness of physical activity and support the development of healthier sports lifestyle patterns. At the same time, shared sports experiences may facilitate communication, strengthen parent–child interaction, and contribute to more positive relational experiences within the family. Overall, evidence suggests that more supportive family sports environments are generally associated with healthier sports lifestyles in adolescents, as well as more harmonious parent–child relationships (Zhang et al., 2024).

Finally, drawing on family systems theory, the family sports environment can be understood as a key ecological component of the family system that may be linked to parent–child relationships through behavioral contexts such as adolescents’ sports lifestyles. In turn, more positive parent–child relationships are typically linked to increased emotional support for adolescents, which is associated with more effective emotional regulation when encountering emotional challenges (Lin and Faldowski, 2023). Taken together, these arguments point to a potential sequential associative mediation pathway in which the family sports environment is associated with adolescents’ sports lifestyles. This, in turn, relates to the quality of parent–child relationships and, ultimately, to adolescents’ emotional regulation. Accordingly, Hypothesis 3 (H3) is proposed: *the parent–child relationship mediates the association between the family sports environment and emotional regulation among Chinese adolescents.*

### 2.4 Mediating effect of sports lifestyle on parent–child relationships

Scholars suggest that a sports lifestyle is associated not only with more positive emotional experiences among Chinese adolescents but also with the dynamics of parent–child relationships that may, in turn, be related to adolescents’ emotional regulation (Li et al., 2024). For example, some studies have reported that regular exercise habits developed through family sports activities are linked to higher-quality parent–child interactions. Moreover, it has been demonstrated that adolescents with healthier sports lifestyles tend to report stronger emotional bonds with their parents (Cohen et al., 2025). From a family systems theory perspective, adolescents’ engagement in regular exercise within a family sports environment may be associated with more frequent and higher-quality parent–child interactions, which in turn may be related to a more supportive emotional climate within the family (Cui et al., 2024). Overall, these findings indicate a close association between sports lifestyles and parent–child relationships. Sports lifestyle patterns may therefore serve as an important behavioral context related to the quality of parent–child interactions.

Furthermore, previous studies have suggested a sequential associative mediating mechanism linking the family sports environment, sports lifestyles, parent–child relationships, and adolescents’ emotional regulation. Evidence indicates that a supportive family sports environment is associated with healthier sports lifestyles among adolescents, which, in turn, are linked to more positive parent–child relationship experiences and more favorable emotional regulation outcomes (Chang and Lai, 2024). In this context, the family sports environment may function as a foundational setting that is related to the development of active sports lifestyles in adolescents. Shared engagement in sports activities may also be associated with frequent and higher-quality parent–child interaction. Collectively, these interconnected factors appear to be linked to adolescents’ emotional regulation capabilities. Accordingly, Hypothesis 4 (H4) is proposed: the family sports environment is associated with adolescents’ emotional regulation through a sequential associative mediating pathway involving sports lifestyle and parent–child relationships.

**Figure 1:**
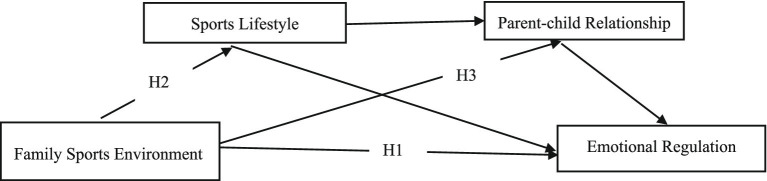
Research framework diagram. H1 represents the direct association between the family sports environment and adolescents’ emotional regulation; H2 represents the mediating role of sports lifestyle; H3 represents the mediating role of parent–child relationships; and H4 represents the sequential mediating association effect of sports lifestyle and parent–child relationships.

In summary, existing literature has identified associative links between the family sports environment and emotional regulation among Chinese adolescents, while also highlighting the potential mediating and sequential mediating roles of sports lifestyles and parent–child relationships in this association. Collectively, these findings provide a solid theoretical foundation for the hypotheses proposed in the present study. Based on the hypotheses outlined above, an integrated conceptual framework was developed, as shown in Figure 1, to examine the interrelationships among the family sports environment, sports lifestyle, parent–child relationships, and adolescents’ emotional regulation.

## 3 Research design and methods

### 3.1 Research subjects

This study adopted a multistage cluster sampling design to improve the representativeness of the sample with respect to both geographic distribution and school type. The sampling framework spanned five major regions of China (Eastern, Southern, Central, Western, and Northeastern China) and covered a total of seven provinces. Specifically, the selected provinces included Jiangsu (Eastern Region), Guangdong (Southern Region), Henan and Hubei (Central Region), Sichuan and Shaanxi (Western Region), and Liaoning (Northeastern Region). Within each prefecture-level city, a stratified sampling strategy was implemented to ensure balanced coverage of schools located in urban core areas, urban–rural transition zones, and county-level regions. Overall, 63 secondary schools were included in the final sample. These schools comprised both public and private institutions, as well as key and general schools, thereby reducing potential sampling bias and ensuring diversity in school characteristics across regions.

### 3.2 Sample size estimation and data collection

This study utilized a structured questionnaire survey comprising the family sports environment scale, the sports lifestyle scale, the parent–child relationship scale, and the adolescent emotional regulation scale. The sample size was determined by integrating theoretical considerations with statistical power analysis. Based on the commonly applied empirical guideline in psychological and educational research that each observed variable requires 10–20 participants, the theoretically required minimum sample size was estimated to be 400–600 individuals. In parallel, *a priori* power analysis using G*Power software indicated that under conditions of a moderate effect size (*d* = 0.5), statistical power (power = 0.80), and a significance level of*α* = 0.05, the recommended sample size range was estimated to be approximately 800 to 1,000 participants. Considering the potential for invalid responses and missing data, a total of 820 questionnaires were ultimately distributed. Data collection was conducted through a decentralized administration strategy. Questionnaires were completed either during after-school hours at school (junior high school: 4:30–5:30 p.m.; senior high school: 5:00–6:00 p.m.) or within the home environment. All survey administrators received standardized training and followed uniform instructions when explaining the study objectives, completion procedures, and confidentiality principles to both participants and their guardians. It is worth noting that informed consent was obtained before participation. The average completion time for each questionnaire was approximately 15 min.

### 3.3 Sample selection and demographic characteristics

Inclusion criteria specified that participants were school-aged adolescents (aged 12–18 years) who reported participation in at least one family sports environment activity per week. This included both parent–child physical activities and individual physical activities occurring within or facilitated by a family-oriented sports context. Following data collection, exclusion criteria were applied. Participants who were not of school age, those reporting insufficient participation in family sports environment activities, questionnaires with ≥5% missing data on core variables, and responses exhibiting obvious logical inconsistencies or pattern-based answering were removed in accordance with established data screening procedures. After data cleaning, 750 valid questionnaires were retained, resulting in a response rate of 94.48%. The mean age of the final sample was 15.26 years (SD = 1.87), comprising 280 junior high school students (37.33%) and 470 senior high school students (62.67%). In terms of gender distribution, 364 participants were males (48.53%), and 386 were females (51.47%), indicating a relatively balanced gender composition. Regarding regional distribution, 118 participants were from the eastern region (15.73%), 145 from the southern region (19.33%), 218 from the central region (29.07%), 182 from the western region (24.27%), and 87 from the northeastern region (11.60%). With respect to residential location, 412 participants (54.93%) lived in urban centers, 198 (26.40%) in urban–rural fringe areas, and 140 (18.67%) in county-level areas. Overall, the sample demonstrated a relatively balanced and structurally reasonable distribution across key demographic variables.

### 3.4 Ethical statement

This study was conducted in strict accordance with the ethical principles outlined in the Declaration of Helsinki. Ethical approval was obtained from the Institutional Review Board of Zhaoqing University (Approval No.: 2025071). Before participation, written informed consent was obtained from all participants and their legal guardians.

### 3.5 Research methods

#### 3.5.1 Psychometric methods

(1) Family Sports Environment Scale.

The Family Sports Environment Scale, developed by Li (2019), was used to assess adolescents’ family sports environments. The scale consists of 9 items and includes three dimensions: Home Sports Facilities and Activity Space, Parental Subjective Support, and Parental Objective Support. Home Sports Facilities and Activity Space assess the availability of sports-related equipment and space within the home (e.g., Item 1: the number of stationary sports equipment items available in the household, such as treadmills, exercise bikes, or multifunctional sit-up boards). Parental Subjective Support reflects emotional and cognitive encouragement from parents (e.g., Item 3: My parents actively inquire about my physical education studies at school). Parental Objective Support measures tangible and financial support for sports participation (e.g., Item 8: My parents provide financial support when I need funds for sports activities, such as paying for sports training or purchasing sports equipment). All items are rated using a 5-point Likert scale. The total scale score is calculated by summing all item responses, with higher scores indicating a higher level of perceived support within the family sports environment and greater access to sports-related resources. The scale has been widely applied in previous studies to assess adolescents’ family sports environments (Hong, 2022), demonstrating good reliability and validity. In the present study, the scale demonstrated excellent internal consistency, with a Cronbach’s *α* coefficient of 0.941. The Kaiser–Meyer–Olkin (KMO) value was 0.962, indicating that the data were highly suitable for factor analysis and supporting the construct validity of the scale. Overall, the instrument demonstrated strong reliability and validity in the current sample.

(2) Sports Lifestyle Scale

The sports lifestyle Scale from the Handbook of Commonly Used Psychological Scales in Sports Science (Zhang and Mao, 2004) was used to assess adolescents’ sports lifestyle. This 30-item scale captures two categories of physical activity motivation, “self-need” and “social extension,” and is structured into six dimensions: Pleasure-seeking, Challenge-seeking, Health-oriented, Fitness-oriented, Learning-oriented, and Social-oriented. The Pleasure-seeking dimension reflects enjoyment derived from physical activity (e.g., Item 1: Participating in physical activities allows me to spend enjoyable time). The Challenge-seeking dimension reflects goal-oriented and performance-enhancing motivation (e.g., Item 6: I participate in physical activities to enhance my physique). The health-oriented dimension reflects perceived health and interpersonal benefits (e.g., Item 11: Exercising or playing sports facilitates interpersonal communication). The Fitness-oriented dimension reflects body management motivations (e.g., Item 18: I participate in physical activities to lose weight or control my weight). The Learning-oriented dimension reflects skill acquisition and exploration motives (e.g., Item 21: I participate in sports to learn and discover new athletic methods). The Social-oriented dimension reflects broader social and transferable skill development (e.g., Item 27: Sports can improve my abilities in other areas, such as aesthetics). All items are rated on a 5-point Likert scale ranging from “1” (strongly agree) to “5” (strongly disagree). In the original scoring method, lower total scores indicate a stronger willingness and motivation to engage in physical activity. For this study, scores were reverse-coded to facilitate statistical analysis and interpretation of results, such that higher reverse-coded total scores reflect a higher level of sports lifestyle engagement and stronger motivation for physical activity among adolescents. All subsequent statistical analyses were conducted using the reverse-coded scores. The scale has been widely applied in previous research to assess adolescents’ sports lifestyle (Jin, 2023), demonstrating good reliability and validity. In the present study, the scale showed excellent internal consistency, with a Cronbach’s *α* coefficient of 0.965. The KMO value was 0.993, indicating that the data were highly suitable for factor analysis and supporting strong construct validity. Overall, the instrument demonstrated excellent reliability and validity in this sample.

(3) Parent–Child Relationship Scale

The Parent–Child Intimacy Scale, originally developed by Buchanan et al. (1991) and revised by Zhang et al. (2011), was used to assess parent–child relationships. The scale consists of 9 items designed to measure the degree of closeness and communication quality between adolescents and their parents (e.g., Item 1: When communicating with your parents, you can freely choose any topic). It employs a five-point Likert scale: 1 = Strongly disagree, 2 = Somewhat disagree, 3 = Neutral, 4 = Somewhat agree, 5 = Strongly agree. The total score is obtained by summing all item scores, with higher scores indicating higher levels of perceived parent–child intimacy and relational closeness. This scale has been widely used in previous research to assess parent–child relationships among adolescents (Hao, 2020), demonstrating good reliability and validity. In the present study, the scale showed high internal consistency, with a Cronbach’s α coefficient of 0.907. The KMO value was 0.951, indicating that the data were highly suitable for factor analysis and supporting strong construct validity. Overall, the questionnaire demonstrated sound reliability and validity in this sample.

(4) Emotion Regulation Scale.

The Emotion Regulation Scale developed by Gross and John (2003) was used in this study to assess adolescents’ emotion regulation abilities. The scale consists of 10 items and is rated on a 7-point Likert scale (1 = Strongly disagree, 2 = Disagree, 3 = Somewhat disagree, 4 = Unsure, 5 = Somewhat agree, 6 = Agree, 7 = Strongly agree). The scale comprises two dimensions: cognitive reappraisal and expressive suppression. Cognitive reappraisal refers to a cognitive strategy in which individuals regulate emotions by changing their interpretation of a situation (e.g., Item 1: “I control my emotions by changing the way I view a situation”). Expression suppression refers to a behavioral strategy in which individuals regulate emotions by inhibiting emotional expression after emotions have arisen (e.g., Item 7: “I control my emotions by not expressing them”) (Gross, 1998; Gross and John, 2003). These two emotion regulation strategies have been shown to differ in their psychological consequences and adaptive value in previous research. In this study, all items from both dimensions were combined to calculate a total emotion regulation score, representing adolescents’ overall self-reported tendency to use emotion regulation strategies in daily life. Higher scores indicate a greater tendency to employ cognitive reappraisal and expressive suppression strategies when regulating emotions. Although the Emotion Regulation Questionnaire (ERQ) is typically used to distinguish between different regulatory strategies, prior studies have also supported the use of the total score as an index of overall emotion regulation tendencies when the focus is on general regulatory capacity rather than strategy-specific differences (Gross and John, 2003; Aldao et al., 2010). The primary objective of the current study is to examine the associational relationship between the family sports environment and adolescents’ overall emotion regulation level, rather than comparative differences between specific regulation strategies. Additionally, to ensure transparency, descriptive statistics and supplementary analytical results for the cognitive reappraisal and expressive suppression are also reported separately. The Emotion Regulation Scale has been widely used in previous research on adolescents’ emotional processes (Larsson et al., 2025), demonstrating good reliability and validity. In this study, the scale showed excellent internal consistency, with a Cronbach’s *α* coefficient of 0.966. The KMO value was 0.976, suggesting that the data were highly suitable for factor analysis and supporting strong construct validity. Overall, the instrument demonstrated excellent reliability and validity in this sample.

#### 3.5.2 Mathematical statistics methods

This study utilized SPSS 26.0 software and the SPSS macro PROCESS developed by Hayes for statistical data analysis. The analytical procedures were conducted as follows: First, SPSS 26.0 was used to perform descriptive statistical analyses of demographic variables and key study variables, including the family physical activity environment, sports lifestyle, parent–child relationship, and adolescents’ emotion regulation. The analyses were conducted to describe the basic characteristics and distributional patterns of each variable. Before conducting group difference tests and correlation analyses, data distributions were examined for normality. In addition, Levene’s test was applied to assess the homogeneity of variances across groups, ensuring that the assumptions for t-tests and one-way ANOVA were satisfied. Additionally, linear relationships among variables were also examined before conducting Pearson correlation analyses. Group comparisons based on demographic characteristics were conducted where appropriate, with statistical significance defined as *p* < 0.05. Effect sizes were reported concurrently alongside significance tests to provide information on the magnitude of observed differences, using Cohen’s d for t-tests and η^2^for ANOVA.

Furthermore, to assess common method bias, Harman’s single-factor test was conducted using exploratory factor analysis on all measurement items. The proportion of variance explained by the first factor was examined to evaluate whether a dominant factor accounted for a substantial proportion of variance in the data. To examine bivariate associations among variables, Pearson correlation analysis was conducted to test relationships among the family physical activity environment, sports lifestyle, parent–child relationship, and adolescents’ emotion regulation. It is worth noting here that emotion regulation was assessed both as a total score across its two dimensions (cognitive reappraisal and expressive suppression), thereby providing a foundation for subsequent mediation analyses. Finally, to examine the hypothesized indirect association pathways, Model 6 of the PROCESS macro proposed by Hayes et al. (2013) was used to construct a chained mediation model. In this model, the family physical activity environment was specified as the independent variable, sports lifestyle and parent–child relationship as sequential mediators, and adolescents’ emotion regulation as the dependent variable. Additionally, gender and age were included as covariates to control for potential confounding effects of demographic factors. All regression analyses and mediation analyses reported unstandardized regression coefficients (B) to ensure interpretability and comparability across pathways.

In addition, mediation effects were tested using the bias-corrected bootstrap method with 5,000 resamples, and 95% confidence intervals were calculated. A mediation effect was considered statistically significant when the confidence interval did not include zero. The bootstrap method does not assume normality of the sampling distribution and therefore provides more robust estimates of indirect effects, serving as an additional sensitivity analysis for the stability of the estimated indirect effects. During data processing, samples with more than 5% missing data on core variables were first excluded. The missing data rates for the remaining variables were low and exhibited patterns consistent with random missingness. Accordingly, mean imputation was applied to the small number of missing values to maintain sample size stability and minimize potential bias introduced by missing data.

## 4 Results

### 4.1 Descriptive statistics analysis of family sports environment, emotional regulation, sports lifestyle, and parent–child relationship

Table 1 indicates that no statistically significant gender differences were observed in the family sports environment, emotional regulation, sports lifestyle, or parent–child relationship (*p* > 0.05). Similarly, no statistically significant age differences were found across these four variables (*p* > 0.05). Male participants showed lower mean scores than female participants across all four variables, including family sports environment, emotion regulation, sports lifestyle, and parent–child relationship. These variables exhibited consistent distributional patterns across different descriptive statistical indicators, providing a preliminary basis for understanding their potential interrelationships and the extent of their bivariate associations.

**Table 1:** Gender Differences in family sports environment, emotional regulation, sports lifestyle, and parent–child relationship.

Variable	Gender	Number (%)	*M*	*SD*	*T*	*p*	Cohen’s d
Variable	Male	364 (48.53%)	34.72	5.098	−0.22	0.826	0.021
	Female	386 (51.47%)	34.8	4.934			
	Total	750	34.76	5.011			
Family Sports Environment	Male	364 (48.53%)	49.5	7.245	−0.59	0.555	0.042
	Female	386 (51.47%)	49.8	6.953			
	Total	750	49.65	7.093			
Emotional Regulation	Male	364 (48.53%)	109.4	12.377	−1.063	0.288	0.080
	Female	386 (51.47%)	110.35	12.055			
	Total	750	109.89	12.213			
Sports Lifestyle	Male	364 (48.53%)	35.58	4.932	−0.498	0.618	0.046
	Female	386 (51.47%)	35.76	4.913			
	Total	750	35.67	4.92			

Cohen’s d values represent the magnitude of effect sizes for gender differences.

Further analyses of effect sizes for between-group differences indicated that gender-related differences were consistently small across all examined variables. Cohen’s d values ranged from 0.02 to 0.08, indicating negligible differences between males and females in the family physical activity environment, emotional regulation, sports lifestyle, and parent–child relationships. Similarly, age-related effect sizes were also minimal, withη^2^ ranging between 0.002 and 0.009. These results indicate that age has limited explanatory power for variations in the studied variables. Therefore, both gender and age were included as covariates in subsequent mediation analyses to account for potential confounding influences of demographic characteristics (see Table 2).

**Table 2:** Age differences in family sports environment, emotional regulation, sports lifestyle, and parent–child relationships.

Variable	Age	Number (%)	*M*	*SD*	*F*	*p*	η^2^
Family sports environment	12	29 (3.87%)	35.79	4.48	0.518	0.795	0.004
	13	86 (11.47%)	34.58	5.51			
	14	165 (22.00%)	34.86	4.88			
	15	195 (26.00%)	34.49	4.91			
	16	169 (22.53%)	34.83	5.13			
	17	78 (10.40%)	34.59	5.11			
	18	28 (3.73%)	35.71	4.62			
	Total	750	34.76	5.01			
Emotional regulation	12	29 (3.87%)	51.24	7.01	0.481	0.823	0.004
	13	86 (11.47%)	49.56	7.39			
	14	165 (22.00%)	49.95	6.45			
	15	195 (26.00%)	49.27	7.58			
	16	169 (22.53%)	49.63	7.20			
	17	78 (10.40%)	49.29	6.71			
	18	28 (3.73%)	50.46	7.08			
	Total	750	49.65	7.09			
Sports lifestyle	12	29 (3.87%)	111.86	9.32	0.267	0.952	0.002
	13	86 (11.47%)	110.23	14.21			
	14	165 (22.00%)	109.70	11.57			
	15	195 (26.00%)	109.88	12.60			
	16	169 (22.53%)	109.91	12.42			
	17	78 (10.40%)	108.85	11.50			
	18	28 (3.73%)	110.86	10.57			
	Total	750	109.89	12.21			
Parent–child relationship	12	29 (3.87%)	36.10	3.96	1.183	0.313	0.009
	13	86 (11.47%)	36.14	5.01			
	14	165 (22.00%)	35.82	4.48			
	15	195 (26.00%)	35.64	4.97			
	16	169 (22.53%)	35.15	5.15			
	17	78 (10.40%)	35.28	5.31			
	18	28 (3.73%)	37.43	4.96			
	Total	750	35.67	4.92			

η^2^ values represent the magnitude of effect sizes for age-related differences.

### 4.2 Common method Bias testing

Given that all data in this study were collected through self-report questionnaires at a single time point, the possibility of common-method bias (CMB) cannot be excluded. To mitigate this concern, several procedural and statistical measures were implemented at both the research design and data analysis stages. At the procedural level, anonymous questionnaires were administered, and participants were explicitly informed that their responses would be used solely for academic research purposes, thereby reducing potential social desirability bias. In addition, item order within the questionnaires was randomized to minimize response patterning effects. Furthermore, reverse-scored items were incorporated into several scales to reduce systematic response bias associated with common-source measurement.

Second, at the statistical diagnostic level, Harman’s single-factor test was conducted as an initial assessment of common method variance (CMV). The unrotated principal component analysis revealed four factors with eigenvalues greater than 1. The first factor accounted for 50.086% of the total variance. This finding indicates that common method variance may be present to a moderate extent; however, it does not provide conclusive evidence of a serious common method bias issue, and its implications should be interpreted with caution in line with prior methodological guidance (Podsakoff et al., 2003). To further examine robustness, subsequent analyses assessed the stability of the core association patterns across alternative model specifications. The results demonstrated that the relationships among the family sports environment, sports lifestyle, parent–child relationship, and adolescents’ emotional regulation remained consistent in both direction and statistical significance. It is worth noting here that no substantive changes were observed that could be attributed to potential common method bias, thereby supporting the stability of the observed associations.

These findings suggest that, although some degree of common method variance may be present, it is unlikely to meaningfully affect the primary association patterns observed in this study. However, the exclusive reliance on a single self-report data source remains a methodological limitation that should be acknowledged. Future research would benefit from adopting longitudinal designs and/or incorporating multi-informant data (e.g., parents and teachers) to further reduce the risk of common method bias and to strengthen the robustness and validity of the observed associations.

### 4.3 Correlation analysis of family sports environment, emotional regulation, sports lifestyle, and parent–child relationship

To examine the bivariate relationships among the study variables, descriptive statistics and correlation analyses were first conducted for the family physical activity environment, sports lifestyle, parent–child relationship, and emotional regulation. Given that the emotional regulation scale consists of two dimensions, cognitive reappraisal and expressive suppression, both the overall emotional regulation score and its two sub-dimensions were included in the correlation analysis. The descriptive statistics and correlation coefficients for each variable are presented in Table 3. The results show that the family physical activity environment is significantly and positively associated with sports lifestyle, parent–child relationships, and the overall emotional regulation score. Further analysis indicates that cognitive reappraisal is significantly and positively associated with the family physical activity environment, sports lifestyle, and parent–child relationships. Similarly, expressive suppression also shows significant positive correlations with these variables. Overall, both emotional regulation strategies exhibit correlation patterns consistent with the total emotional regulation score, thereby providing empirical support for subsequent analyses of indirect associations.

**Table 3:** Descriptive statistics and correlation analysis for each variable.

Variable	M ± SD	Family sports environment	Sports lifestyle	Parent–Child relationship	Emotional regulation	Cognitive reappraisal	Expressive suppression
Family sports environment	34.764 ± 5.011	1					
Sports lifestyle	109.892 ± 12.213	0.803**	1				
Parent–Child relationship	35.675 ± 4.920	0.747**	0.799**	1			
Emotional regulation	49.655 ± 7.093	0.698**	0.757**	0.752**	1		
Cognitive reappraisal	24.827 ± 3.573	0.689**	0.746**	0.745**	0.983**	1	
Expressive suppression	24.828 ± 3.639	0.685**	0.744**	0.734**	0.984**	0.334	1

*N* = 750, **p < 0.01.

### 4.4 Testing the mediating effect of family sports environment on emotional regulation

With the family physical activity environment designated as the independent variable, sports lifestyle and parent–child relationship as mediating variables, and adolescents’ emotional regulation as the dependent variable, a serial mediation analysis was performed using the PROCESS macro for SPSS developed by Hayes. Model 6 was applied, and a non-parametric bootstrap procedure with bias correction was adopted to test the indirect associations. A total of 5,000 bootstrap resamples were generated to construct 95% confidence intervals. A mediating effect was considered statistically significant when the confidence interval did not include zero. The regression results are presented in Table 4. The total effect of the family sports environment on adolescents’ emotional regulation was significant (*B* = 0.988, *t* = 26.811, *p* < 0.001). After including both mediators in the model, the direct effect remained statistically significant (*B* = 0.207, *t* = 3.829, *p* < 0.001). Regarding the specific pathways, the family sports environment is significantly and positively associated with sports lifestyle (*B* = 1.957, *t* = 37.969, *p* < 0.001) and parent–child relationship (*B* = 0.292, *t* = 8.445, *p* < 0.001). In turn, sports lifestyle is significantly and positively associated with both parent–child relationship (*B* = 0.226, *t* = 15.931, *p* < 0.001) and adolescents’ emotional regulation (*B* = 0.203, *t* = 8.400, *p* < 0.001). Additionally, parent–child relationship is significantly and positively associated with adolescents’ emotional regulation (*B* = 0.524, *t* = 9.175, *p* < 0.001). Taken together, all pathway coefficients reached statistical significance, indicating that the family sports environment is statistically associated with adolescents’ emotion regulation both directly and indirectly through sports lifestyle and parent–child relationship. These findings provide statistical support for Hypothesis H1 in terms of observed associations. It should be noted that all reported coefficients are unstandardized regression coefficients (B). Given differences in measurement scales across variables, values exceeding 1 are statistically acceptable and do not affect the interpretation or validity of the model results (Deegan, 1978) and does not affect the interpretation or validity of the model results.

**Table 4:** Regression analysis of the chain mediation model between family sports environment and emotional regulation in Chinese adolescents.

Variable	Family sports environment		Parent–Child relationship		Emotional regulation	
	*B*	*t*	*B*	*t*	*B*	*t*
Family sports environment	1.957	37.969	0.292	8.445	0.207	3.829
Sports lifestyle	—	—	0.226	15.931	0.203	8.400
Parent–Child relationship	—	—	—	—	0.524	9.175
*R* ^2^	0.645		0.67		0.64	
F	1441.675		765.464		464.122	

B represents unstandardized regression coefficients; *t* values indicate the corresponding significance tests. All *p*-values are less than 0.001.

**Figure 2:**
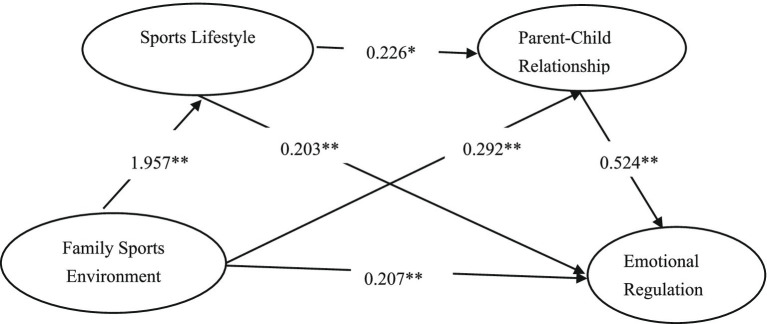
Chain mediation model of sports lifestyle (M1) and parent–child relationship (M2) mediating between family sports environment (X) and adolescent emotional regulation (Y).

Further mediation analyses were conducted using the bootstrap method, and the results are presented in Table 5. The total indirect effect of the family sports environment on adolescents’ emotional regulation was 0.781, with a 95% bootstrap confidence interval of [0.689, 0.878]. As the confidence interval does not include zero, this finding indicates that both a physical activity-oriented lifestyle and parent–child relationship significantly account for the indirect association between family sports environment and emotional regulation. In terms of effect decomposition, the direct effect accounted for 20.95% of the total effect, whereas the combined indirect effect accounted for 79.05%, indicating that the observed association is primarily reflected through indirect pathways. More specifically, three distinct indirect effects were identified. First, the indirect pathway through physical activity lifestyle (“family sports environment → sports lifestyle → emotional regulation”) yielded an effect size of 0.396, with a 95% confidence interval of [0.300, 0.497]. Because the interval does not include zero, this pathway is statistically significant, indicating that a sports lifestyle is statistically associated with the link between the family sports environment and emotion regulation. This indirect path accounts for 40.08% of the total effect, providing support for Hypothesis H2 in terms of observed associations. Second, the indirect pathway through parent–child relationship (“family sports environment → parent–child relationship → emotional regulation”) produced an effect size of 0.232, with a 95% confidence interval of [0.178, 0.292]. This result also excludes zero, indicating a statistically significant indirect association involving the parent–child relationship. This pathway accounts for 23.48% of the total effect, providing support for Hypothesis H3 in terms of observed associations. Third, the sequential mediation pathway (“family sports environment → sports lifestyle → parent–child relationship → emotional regulation”) yielded an effect of 0.153, with a 95% confidence interval of [0.109, 0.206]. As the confidence interval does not include zero, this indicates a statistically significant sequential indirect association involving sports lifestyle and parent–child relationship. This indirect pathway accounts for 15.49% of the total effect, providing support for Hypothesis H4 in terms of observed associations (see Figure 2).

**Table 5:** Bootstrap test of the chain mediation effect of sports lifestyle and parent–child relationship between family sports environment and adolescents’ emotional regulation.

Effect type	Effect B	Boot SE	95% CI lower	95% CI upper	Proportion of total effect
Total effect	0.988	0.037	0.916	1.061	100%
Direct effect	0.207	0.054	0.101	0.314	20.95%
Family sports environment → Sports lifestyle → Emotional regulation	0.396	0.05	0.300	0.497	40.08%
Family sports environment → Parent–child relationship → Emotional regulation	0.232	0.029	0.178	0.292	23.48%
					100 percent 20.95 percent 40.08 percent 23.48 percent 15.49 percent 79.05 percent
Family sports environment → Sports lifestyle → Parent–child relationship → Emotional regulation	0.153	0.025	0.109	0.206	15.49%
					100 percent 20.95 percent 40.08 percent 23.48 percent 15.49 percent 79.05 percent
Total indirect effect	0.781	0.048	0.689	0.878	79.05%

Effect B represents the unstandardized indirect effect estimate; Boot SE indicates bootstrap standard error; CI refers to the bias-corrected 95% confidence interval; Bootstrap samples = 5,000.

## 5 Discussion

### 5.1 Family sports environment and adolescent emotional regulation

The findings of this study demonstrate a significant positive association between the family physical activity environment and emotional regulation among Chinese adolescents, which is broadly consistent with existing research (Sui and Huang, 2025). Prior research has consistently highlighted that family-related physical activity factors, such as parental modeling of exercise, shared family participation in physical activity, and overall home-based activity support, are positively associated with adolescents’ psychological functioning and emotional experiences (Morbée et al., 2023). From a broader perspective, previous studies have also documented meaningful associations among social support, physical activity behaviors, and mental health outcomes. For instance, Ren and Li (2020) identified a significant relationship between perceived social support and adolescents’ physical activity levels. Similarly, Cao et al. (2022) and Lu et al. (2020) reported associations between physical activity patterns, sedentary behavior, sleep quality, and emotional outcomes in adolescents. In addition, Chi and Wang (2022) found that levels of sports participation were not significantly associated with symptoms of depression and anxiety. Collectively, these studies converge in suggesting a consistent pattern of associations between physical activity-related contexts and adolescents’ emotional and mental health status, providing a broader empirical foundation for the present findings.

However, these findings should be interpreted with caution, given the limitations of the study design. First, this study adopted a cross-sectional survey design in which all variables were collected at a single time point through adolescent self-reports. As a result, the findings primarily reflect statistical associations among variables and do not support inferences about temporal ordering or causal relationships. Accordingly, the pathways specified in the chained mediation model should be understood as theoretically informed statistical associations rather than evidence of a confirmed causal sequence among the variables. Second, several alternative explanations should be acknowledged. On one hand, adolescents with stronger emotional regulation abilities may be more likely to engage in family-based physical activities or to perceive the family physical environment more positively. Thus, the observed associations may partly reflect the influence of individual emotional characteristics on perceptions of the family context. On the other hand, broader contextual factors such as overall family functioning, parenting styles, and socioeconomic conditions may be associated with both the family sports environment and adolescents’ emotional states. However, these factors were not systematically incorporated in the present model. Therefore, the current findings are best interpreted as a pattern of associations among variables rather than evidence of unidirectional or causal relationships.

Furthermore, this study combined cognitive reappraisal and emotional suppression into a composite measure of emotional regulation for analysis. Although this approach captures an individual’s overall self-reported tendency toward emotion regulation, existing research suggests that these two strategies differ in their psychological and behavioral correlates. Cognitive reappraisal is typically associated with more adaptive psychological outcomes, whereas the effects of emotional suppression are more context-dependent and can vary across situations (Gross and John, 2003). Accordingly, combining the two strategies into a single indicator may partially obscure meaningful differences in their distinct associations with family and behavioral contexts. Future research should therefore differentiate between these emotion regulation strategies to further clarify their unique associations with family sports environments and related psychosocial factors. Overall, building on existing literature, this study integrates the family physical activity environment, sports lifestyle, and parent–child relationships within a unified analytical framework. In doing so, it identifies a pattern of statistical associations among these variables and adolescents’ emotional regulation. However, given the cross-sectional design and reliance on self-reported data, the findings require further validation. Future studies should employ longitudinal designs, multi-informant data sources, and behavioral measures to more robustly examine the patterns of associations among these variables.

### 5.2 Mediating role of sports lifestyle

The results indicate that a sports lifestyle accounts for part of the association between the family sports environment and adolescents’ emotional regulation, which is consistent with previous research. A sports lifestyle is generally defined as a relatively stable pattern of adolescent behavior, including the frequency of sports participation, types of activities, and attitudes toward sports (Carter et al., 2023). Existing studies suggest that such behavioral patterns are associated with adolescents’ emotional states and broader psychological adjustment (Zhang et al., 2024). At the family level, evidence shows that parental attitudes toward physical activity, availability of family physical activity resources, and shared participation in sports are often significantly associated with adolescents’ levels of physical activity engagement (Zheng et al., 2025; Jiang and Xiao, 2024). From a theoretical perspective, Self-Determination Theory posits that sustained behavioral engagement is more likely when individuals experience autonomy, competence, and relatedness (Liu et al., 2025). Within this framework, a supportive family physical activity environment may be theoretically linked to the development and maintenance of adolescents’ sports lifestyles through the reinforcement of these psychological needs.

However, these relationships should be interpreted with caution due to methodological considerations. Given that the data were collected through self-report questionnaires at a single time point, the observed associations may also reflect individual subjective perceptions. For instance, adolescents with more stable positive emotional states may be more likely to report healthier or more active lifestyles. Furthermore, the relationships among physical activity participation, emotional experiences, and family interactions are likely to be more complex and reciprocal than captured in the present model. Therefore, future research should employ longitudinal designs and multi-source data to examine the dynamic associations among these variables.

### 5.3 Mediating role of parent–child relationship

The results of this study indicate that the parent–child relationship accounts for part of the association between the family sports environment and adolescents’ emotional regulation. Existing research consistently highlights a close link between the quality of parent–child relationships and adolescents’ emotional experiences (Aneesh et al., 2024). Parent–child relationships are typically conceptualized in terms of emotional communication, trust, and patterns of daily interaction (Steinberg, 2001; Laursen and Collins, 2009). Within these interactions, parental emotional responsiveness and support often coincide with adolescents’ emotional expressions and regulation processes (Kerr et al., 2019). Additionally, prior studies suggest that shared family activities provide important contexts for parent–child interaction, with physical activity representing a common form of such engagement (Koga et al., 2023). These shared activities may increase opportunities for communication between parents and children, thereby being associated with stronger relational quality. Accordingly, family physical activity contexts have been associated with improved parent–child relationship quality in several studies (La Rosa et al., 2025; Cohen et al., 2025).

However, these findings should be interpreted in light of the specific research design. On the one hand, the parent–child relationship data in this study were based on adolescents’ self-reports, and these evaluations may have been influenced by their current emotional states or individual cognitive styles. On the other hand, the parent–child relationship may also reflect broader family interaction patterns, such as general communication styles and parenting practices, which are themselves likely to be associated with adolescents’ emotional states. Accordingly, the observed associations may partly capture shared method variance and family-level processes rather than isolated relational effects. Therefore, future research should incorporate multiple data sources, such as parent reports, teacher evaluations, and behavioral observation methods, to more robustly validate these associations.

### 5.4 Mediating effect of sports lifestyle and parent–child relationship

The findings further suggest the existence of a sequential indirect association pathway involving sports lifestyle and parent–child relationships between the family sports environment and adolescents’ emotional regulation. Previous research has indicated that adolescents’ sports participation is associated with family interaction patterns, as shared physical activities often increase opportunities for parent–child engagement (Meerits et al., 2023; Siu and Lo, 2020; Jiang and Xiao, 2024). Within this context, both a sports-oriented lifestyle and parent–child interaction patterns may exhibit interconnected and mutually reinforcing associations. Additionally, prior studies have shown that sports participation within the family context, parent–child interaction, and adolescents’ psychological outcomes often form a complex multivariate association structure rather than isolated relationships (Zeng et al., 2025; Yang et al., 2025; Cui et al., 2024). The present results statistically reflect a similar pattern, suggesting a sequential association pattern among the family sports environment, sports lifestyle, parent–child relationships, and emotional regulation. However, it is important to emphasize that this chain mediation pathway is derived primarily from cross-sectional data and therefore does not reflect the actual temporal sequence among variables. Moreover, the observed relationships may be jointly influenced by other family-level factors and individual factors. For example, the overall family interactive climate or adolescents’ personality traits may simultaneously be associated with physical activity engagement, parent–child relationship quality, and emotional regulation outcomes. Therefore, future research should further examine these dynamic mechanisms using longitudinal designs or experimental approaches to better clarify potential directional processes.

Overall, this study reveals a multivariate pattern of associations among the family context, behavioral patterns, and relational structures. This pattern suggests that understanding adolescents’ emotional regulation cannot rely solely on individual behaviors or isolated family factors. Instead, these variables are more likely to co-occur within an interconnected configuration of context, behavior, and relationships. Accordingly, explanatory approaches focused on single determinants may be insufficient to capture the complexity of these processes. Future research should therefore examine these interdependent association variables within a multi-level analytical framework.

## 6 Practical significance

This study systematically examines the influence of the associational pattern of family sports environments on adolescents’ emotional regulation through the statistical pathways involving sports lifestyle and parent–child relationship, using these as core variables. It offers several important practical implications. First, the findings provide theoretical support for adolescent mental health education and family-based physical activity guidance. They highlight the observed association between the family sports environment in supporting adolescents’ emotional regulation abilities, suggesting that parents should actively cultivate a positive family sports atmosphere, strengthen role modeling in physical activity participation, and support parent–child engagement in sports to establish a supportive foundation for adolescents’ emotional well-being. Second, the results reveal the statistically identified indirect associations involving sports lifestyle and parent–child relationship in this process. This provides practical guidance for the design of family-based physical activity interventions and the optimization of parent–child interaction patterns, supporting the implementation of youth-centered family sports approaches and enhancing the emotional and educational value of shared physical activity. Finally, the study contributes evidence-based insights for supporting adolescents’ physical and mental health, supporting healthy lifestyle development, and strengthening emotional regulation capacities. In this way, it may inform improvements in adolescent mental health education systems, support the advancement of high-quality family sports development, and contribute to strengthening the psychological well-being of adolescents.

## 7 Research limitations and future directions

Although this study examined and analyzed the associations among the family physical activity environment, physical activity lifestyle, parent–child relationships, and adolescents’ emotional regulation based on existing literature, several limitations should be acknowledged. First, regarding data sources, the study primarily relied on adolescents’ self-report questionnaire data. Although several procedural measures were applied to reduce social desirability bias, including anonymous responses, random item ordering, and reverse-coded items, and were implemented during the study design phase to reduce social desirability bias, and although Harman’s single-factor test was conducted to assess common method variance, the first factor accounted for 50.086% of the variance, This suggests that common method variance may still be present to some extent. Such bias may inflate observed associations among variables and influence the estimation of their relationships. Second, the study employed a cross-sectional design in which all variables were measured at a single time point. As a result, the findings primarily reflect statistical associations rather than temporal ordering or causal relationships. The proposed chain mediation model is therefore grounded in theoretical assumptions, and its implied pathways require further validation through longitudinal or experimental research designs. Third, the core constructs were measured exclusively using self-report scales, without incorporating behavioral observations or objective indicators. This reliance on subjective assessments may introduce cognitive and perceptual bias, which could affect the robustness of the findings and the stability of the observed associations. Finally, in terms of sample characteristics, the study was conducted primarily among adolescents from specific regions. Limited representation of urban–rural differences, socioeconomic diversity, and varied family structures (e.g., single-parent or multigenerational households) constrains the generalizability of the findings.

Given these limitations, future research could be extended in several directions. First, in terms of research methods, a multi-source data collection strategy should be adopted. This may include integrating parent and teacher reports, observational data on parent–child physical activity interactions, and objective indicators such as wearable devices or digital activity logs. Such an approach would help reduce bias resulting from single-informant, self-report measurements and improve data validity. Moreover, regarding research design, future studies should employ longitudinal tracking, as well as experimental or quasi-experimental designs, to more systematically examine the dynamic associations among family physical activity contexts, behavioral engagement, and adolescents’ emotional development. This would allow for stronger inferences regarding temporal ordering and potential directional processes, rather than causal mechanisms. Furthermore, in terms of sampling strategy, future research should expand to multi-regional or multi-center investigations. Incorporating adolescents from diverse socioeconomic backgrounds and varied family structures would improve the representativeness and generalizability of the research findings. Finally, future research could also incorporate additional contextual and ecological variables into analytical models. These may include parenting styles, peer influences, school physical education environments, and family socioeconomic status. Integrating these factors would provide a more comprehensive understanding of the multifactorial associations between family physical activity contexts and adolescents’ emotional development.

## 8 Conclusion

This study confirms a significant positive association between the family sports environment and adolescents’ emotional regulation abilities in China and further identifies a chain indirect association pathway involving sports lifestyle and parent–child relationships. The findings indicate that the family sports environment is both directly and indirectly associated with adolescents’ emotion regulation abilities through sequential pathways involving sports lifestyle and parent–child relationship quality. These results contribute to the literature on adolescent mental health and family sports by extending understanding of how family environmental factors are associated with psychological development and by refining the relevant theoretical framework. Practically, the findings suggest that families should actively cultivate a supportive sports-oriented atmosphere, strengthen high-quality parent–child physical activity interactions, encourage sustained sports participation among adolescents, and provide consistent emotional responsiveness within daily family life. Through enhancing engagement in physical activity and strengthening parent–child emotional bonds, families may play an important role in supporting adolescents’ emotional well-being. Despite limitations related to sampling and research design, these findings provide meaningful theoretical insights and practical implications for adolescent mental health education, family sports encouragement, and the optimization of parent–child interaction practices. Future research should employ longitudinal designs and multi-method data collection approaches to further examine the dynamic patterns of associations among these variables, thereby improving both the scientific robustness and practical applicability of the findings.

## Data Availability

The raw data supporting the conclusions of this article will be made available by the authors, without undue reservation.
